# Improved Estimation of Bio-Oil Yield Based on Pyrolysis Conditions and Biomass Compositions Using GA- and PSO-ANFIS Models

**DOI:** 10.1155/2021/2204021

**Published:** 2021-10-05

**Authors:** Zhimin Li, Deyin Zhao, Linbo Han, Li Yu, Mohammad Mahdi Molla Jafari

**Affiliations:** ^1^Research Institute of Petroleum Engineering and Technology, Sinopec Northwest Oilfield Company, Urumqi 830011, China; ^2^College of Health Science and Environmental Engineering, Shenzhen Technology University, Shenzhen 518118, China; ^3^Department of Petroleum Engineering, Ahwaz Faculty of Petroleum Engineering, Petroleum University of Technology (PUT), Ahwaz, Iran

## Abstract

This paper incorporates the adaptive neurofuzzy inference system (ANFIS) technique to model the yield of bio-oil. The estimation of this parameter was performed according to pyrolysis conditions and biomass compositions of feedstock. For this purpose, this paper innovates two optimization methods including a genetic algorithm (GA) and particle swarm optimization (PSO). Primary data were gathered from previous studies and included 244 data of biodiesel oils. The findings showed a coefficient determination (*R*^2^) of 0.937 and RMSE of 2.1053 for the GA-ANFIS model, and a coefficient determination (*R*^2^) of 0.968 and RMSE of 1.4443 for PSO-ANFIS. This study indicates the capability of the PSO-ANFIS algorithm in the estimation of the bio-oil yield. According to the performed analysis, this model shows a higher ability than the previously presented models in predicting the target values and can be a suitable alternative to time-consuming and difficult experimental tests.

## 1. Introduction

Bioenergy is by far the most successful and sustainable future path [1]. The primary source of energy today is fossil fuels that have enormously negative environmental consequences, causing many issues around the world [2]. Thankfully, biomass energy with neutral carbons is a viable means of addressing both energy needs and environmental issues [3, 4]. In addition, a massive quantity of potential supplies is analyzed each year [5–7]. Thermochemical and biochemical conversions are the procedures appropriate for efficient biomass consumption, which are currently being researched. Thermochemical conversion has drawn the attention of researchers in recent years due to the elevated level of conversion performance besides minimal costs [8]. Pyrolysis is a thermochemical conversion method that involves heating feedstock in an inert environment or oxygen-deficient atmosphere to generate biochar, bio-oils, and noncondensable gas [9]. Bio-oils are liquid substances that typically contain over 350 chemicals, including several materials in short supply [10]. Furthermore, provided bio-oil is properly improved, it has the potential to be a viable alternative energy source, compared to fossil fuels. Moreover, the bio-oil hydrogen content typically represents the heating efficiency and chemical composition (i.e., bio-oil efficiency), whereas the yield refers to the amount of bio-oil. The quality of bio-oils and their quantity are primarily determined by biomass feedstock as well as pyrolysis circumstances [11]. Using proximate and ultimate analyses can generally produce data on various biomasses. The proximate analysis could be used to establish the concentration of fixed carbon, ash, and volatile material. The organic compartment in the biomass is determined by the amount of fixed carbon and volatile material, whereas the ash usually reflects inorganic salts. In the meantime, the content of basic elements (i.e., C−H−N−O) is primarily defined by ultimate analysis. The pyrolysis circumstances include the temperature, the size of the particles, heating rate, and residence period during the pyrolysis process. As a result, several inquiries have been launched in the field of study. Akhtar and Amin reported that the intermediate pyrolysis temperature (500−550°C) normally increased the yield of bio-oil to the maximum extent [12]. Gholizadeh and colleagues reported that the production of bio-oil from herbaceous biomass was smaller compared with that obtained from woody biomass. In addition, the amount of hydrogen in bio-oil was usually greater than in feedstock [13]. Chiodo et al. realized that bio-oils extracted from woody biomass possess more amounts of hydrogen than that from algae, resulting in a greater thermal output [14]. Nonetheless, the relationship between bio-oil characteristics containing biomass compositions and operational parameters remains unclear, due to experimental and financial constraints. The linear regression approach is the most commonly used method for detecting variable correlation. Li et al. investigated the relationship between the distribution of bio-oil compounds and feedstock features using linear regression [15]. Oasmaa et al. established the association between the organic and ash amounts [16]. While the output of linear regression can be undesirable in the presence of a nonlinear association between variables, after the emergence of artificial intelligence, several new approaches were applied to conventional studies and yielded suitable results [17–24]. Cao et al. used a least-squares support vector machine (LM-SVM) and an artificial neural network (ANN) to reliably estimate biochar yields from cattle manure [25]. Sun et al. used the Levenberg Marquardt ANN approach to specifically assess the significance of every variable for the gas yield [26]. Satisfactorily, the ANN method was used by Naqvi et al. to research the mechanism of the reaction according to data related to copyrolysis thermal decomposition [27]. SVM and ANN models were developed by Xing et al. to thoroughly make an estimation of the biomass heating rate by proximate and ultimate analyses [28]. The entire models aided researchers in evaluating a specific outcome without running tests, besides expanding their understanding of the biomass pyrolysis mechanism. Nonetheless, these models were mostly concerned with estimation, leaving the finer knowledge to be retrieved. Random forest (RF) is defined as an ensemble study approach focused on tree predictors that can solve regression and classification problems [29]. Zhu et al. skillfully and accurately predicted biochar yields by the use of the RF approach, and, at the same time, they established associations between biochar production, biomass structural details, and pyrolysis circumstances [30]. Using the RF model, Xing et al. accurately predicted the biomass chemical composition from the ultimate analysis [31]. Due to the properties of the ensemble analysis, the RF approach can achieve higher training rates and superior productivity than other estimation techniques. Further, high-dimensional properties and feature correlations can be addressed and established using the RF procedure.

In this paper, for the first time, attempts have been made to estimate models using the two models GA- and PSO-ANFIS. For this purpose, first, the relevant input data affecting the output parameter were collected, and then, this issue was modeled. Finally, in order to evaluate the strength of these models, various statistical analyses were used.

## 2. Theory

### 2.1. The Adaptive Neurofuzzy Inference System

As a general guideline, a Takagi-Sugeno fuzzy rule and input-output variables form the basis of an adaptive neurofuzzy inference system (ANFIS). Generally, an adaptive neurofuzzy inference system (ANFIS) involves input-output variables and a Takagi-Sugeno fuzzy rule [32–34]. An adaptive, multilayer, and feed-forward network described by ANFIS can be simplified by expressing it as two inputs (*x*, *y*) and one output (*z*). The ANFIS model is an adaptive, multilayer, and feed-forward network that, for the sake of simplicity, can be expressed with two inputs (*x*, *y*) and one output (*z*). Following that, two different if-then fuzzy rules are set for a first-order Sugeno fuzzy model to determine the matching principle. Next, the matching principle is set with two different if-then fuzzy rules for a first-order Sugeno fuzzy model:

(1) $$\begin{array}{c} \text{Rule }1:\text{if }x\text{ is }A_{1}\text{ and }y\text{ is }B_{1},\text{then }Z_{1}=P_{1}x+q_{1}y+r_{1},\\ \text{Rule }2:\text{if }x\text{ is }A_{2}\text{ and }y\;\text{is }B_{2},\text{then }Z_{2}=P_{2}x+q_{2}y+r_{2}. \end{array}$$

This equation evaluates entries through linguistic B1 as well as A1 variable entries of this equation are evaluated through linguistic A1 and B1 variables. In order to calculate the outcome of every rule, the inputs with the constant term (*r*) can be linearly combined. The results of each rule can be calculated using a linear combination of the inputs with the constant term (*r*). There are five layers in ANFIS architecture, with the first layer undergoing fuzzification to map the *x* and *y* variables (inputs) into fuzzy sets (that is, A1, A2, B1, and B2). The membership grades for square nodes are determined by node functions. Each square node generates membership grades using node functions. An alternative to linguistic labels (for example, high and low) uses symbols such as A and B. Instead of linguistic labels (e.g., high and low), characters such as A and B are used. The labels are classified according to their membership functions; for example, different membership functions serve to characterize the labels, e.g., the sigmoid, triangular, and generalized bell functions. Various sets of fuzzy inputs and firing strength are used in layer two. There exist combinations of fuzzy sets of inputs in layer two and the use of firing strength. The fuzzy conjunction “and” is successfully utilized by the G-norm operator to locate the output in this layer. In this layer, a G-norm operator successfully performs the fuzzy conjunction “and” to find the output. Calculation of the *i*th rule ratio is done at the third layer. The third layer involves calculating the ratio of the *i*th rule. At the fourth layer, a function of the Sugeno fuzzy rule is multiplied by the output of the three previous layers. Next, in the fourth layer, the output of the three former layers is multiplied by the function of the Sugeno fuzzy rule. One node in the fifth and last layer is responsible for computing and summarizing each rule output from the previous layer. The fifth and final layer, which contains a single node, involves the summation and calculation of the outputs associated with each rule from the fourth layer. The next step is the application of the weight average method to perform defuzzification. Next, the weight averaged approach is incorporated to carry out the defuzzification process. During this process, fuzzy outputs are transformed into crisp ones. This process results in a crisp output by transforming the fuzzy outputs. ANFIS parameters fall into the consequent and the premise parts depending on whether linear or nonlinear parameters are used. ANFIS parameters can be classified into two categories: linear parameters in the consequent part and nonlinear in the premise part. The parameters can be optimized by gradient descent or steepest descent, among other methods. These parameters can be optimized through a variety of methods, such as gradient descent and steepest descent methods. However, the hybrid learning method is much more effective. Yet, much higher efficiency can be achieved through the hybrid learning method [32].

### 2.2. Particle Swarm Optimization (PSO)

The fundamental knowledge for PSO came from configuring natural populations (e.g., birds) [35, 36]. In PSO, the optimizing problem is the particle and the answer is obtained through generation update. The swarm denotes the total number of particles [37]. This way, the particle is considered as an individual and the swarm as a population. The above expressions also exist in most other evolutionary methods, such as genetic algorithms (GA) [38]. However, the evolutionary type operators (e.g., mutations) do not exist in PSO [37]. Particles, during the process of finding the optimal answer, search for the problem domain and, in the meantime, are affected by their topological neighborhoods (e.g., queen, physical, and social) [39]. Equation (2) calculates the *i*th particle velocity. In this equation, *vi* (*t*) depicts the velocity vector and *xi* (*t*) represents the position vector [38, 40].

(2) $$\begin{array}{c} v_{id}\left(t+1\right)=c_{1}r_{1}\left(p_{\text{best}},id\left(t\right)\right)-\left.x_{id}\left(t\right)\right)+{wv}_{id}\left(t\right)+c_{2}r_{2}\left(g_{\text{best}},id\left(t\right)-x_{id}\left(t\right)\right),d=1,2,\cdots ,D. \end{array}$$

Additionally, *p*_best_, *id* represents the best position, *w* is representative of the inertia's weight, and *g*_best_, *id* represents the best global position of the *i*th particle. Random coefficients are represented by *r*_1_ and *r*_2_, together with the degrees of learning by *c*_1_ and *c*_2_ [41]. In Equation (2), the first term is a cognitive element directing the movements in particles and the second term denotes the previous movement route memory, and finally, the last term serves to assess the particle action in comparison with its neighborhood [35, 38]. Equation (3) provides an integrating process that helps calculate the position vector.

(3) $$\begin{array}{c} x_{id}\left(t+1\right)=x_{id}\left(t\right)+v_{id}\left(t+1\right),d=1,2,\cdots ,D. \end{array}$$

### 2.3. Genetic Algorithm (GA)

An evolutionary heuristic algorithm such as GA imitates the natural process of evolution to optimization. To resolve optimization issues, the GA can be used to calculate the best solution. Holland developed GA when he utilized a common functional framework in 1975 [42, 43]. The development of the algorithm was inspired by Darwin's natural selection theory. In fact, the GA method makes it possible to renew the genetic behavior observed in a biological population. Generally, chromosomes, also known as individuals, are referred to as candidate answers to a particular problem in the GA which typically comprises a linear array of genes. By randomly using the generated design populations, the search process is started. The search process does not require the definition of starting points because it is iterative. In the GA technique, the multiplication from one generation to the subsequent generation is performed by three operators during the optimization. When GA takes into account the theory of a greater chance of survival in order to generate design solutions, the “Selection” operator is the first operand. At all stages of the process of selection, these solutions must be compatible with their environment. “Crossover” is the second operator, and it triggers mating among the biological populations. Crossover operators ensure that fitting surviving characteristics are transferred from the current to subsequent populations. With this method, it is more likely that arbitrarily surviving will be included in the population. “Mutation” is the third operator responsible for creating heterogeneity in the characteristics of the population. According to Dutch (1975), Hasan, and Cohanim et al. (2005), mutation operator performs the worldwide search in the search space and also does not allow the genetic algorithm located in local minima.

## 3. Data Bank

From previous researches, a total of 244 samples involving biodiesel oil yield on the basis of pyrolysis conditions and biomass compositions of feedstock were gathered [44]. The samples were categorized into a training cluster (183 samples) and a test group (61 samples).

## 4. Model Results

Methods such as STD, MSE, RMSE, MRE %, and *R*^2^ were used to analyze the obtained yield values (Table 1) against real data. The statistical parameters were derived from the formulas as follows [45–49]:

(4) $$\begin{array}{c} R^{2}=1-\frac{\sum_{i=1}^{n}{\left[x_{i}^{\text{sim}}-x_{i}^{\exp}\right]}^{2}}{\sum_{i=1}^{n}{\left[x_{i}^{\text{sim}}-x^{m}\right]}^{2}},x_{m}=\frac{\sum_{i=1}^{n}x_{i}^{\exp}}{n}, \end{array}$$

(5) $$\begin{array}{c} \text{MRE}=\frac{1}{N}\overset{N}{\underset{i=1}{\sum }}\frac{\left|x_{i}^{\exp}-x_{i}^{\text{sim}}\right|}{x_{i}^{\exp}}, \end{array}$$

(6) $$\begin{array}{c} \text{MSE}=\frac{1}{n}\overset{n}{\underset{i=1}{\sum }}{\left(x_{i}^{\exp}-x_{i}^{\text{sim}}\right)}^{2}, \end{array}$$

(7) $$\begin{array}{c} \text{RMSE}={\left(\frac{1}{N}\overset{N}{\underset{i=1}{\sum }}{\left(x_{i}^{\exp}-{Yx}_{i}^{\text{sim}}\right)}^{2}\right)}^{0.5}, \end{array}$$

(8) $$\begin{array}{c} \text{STD}=\sqrt{\overset{n}{\underset{i=1}{\sum }}\left(\frac{{\left(x_{i}^{\text{sim}}-x_{m}\right)}^{2}}{n}\right).} \end{array}$$

In Equations (4)–(8), the character *x*_*i*_^exp^ denotes the experimental target value, and *x*_*i*_^sim^ represents the simulated value. The number of experimental data is shown by *n*. Table 1 displays the data calculated for these parameters. A more favorable model has smaller RMSE, MRE, MSE, STD, and larger *R*^2^. As is observed, the GA-ANFIS method is not as precise in training, testing, and total datasets compared with the PSO-ANFIS model (see Table 1).

**Figure 1:**
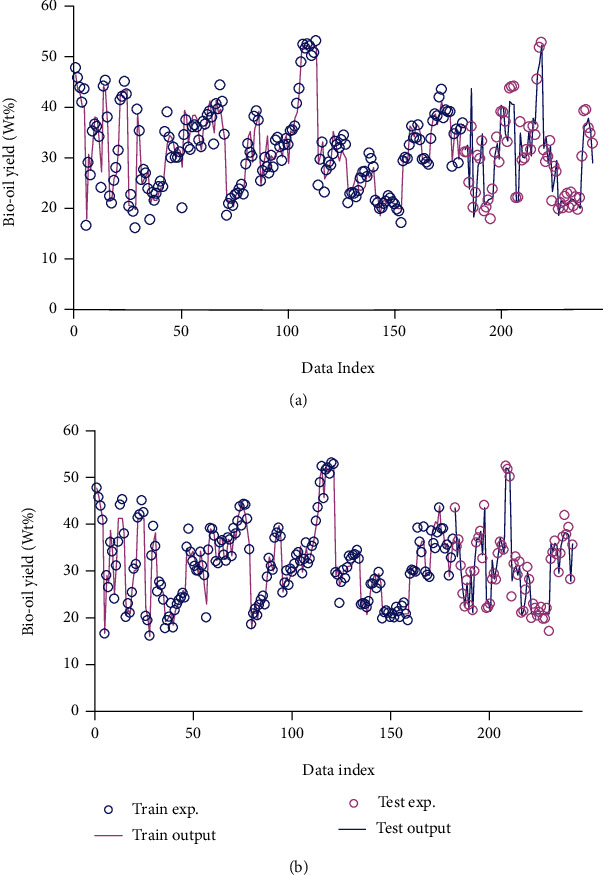
Simultaneous and visual comparison between actual and modeled output data for models (a) GA-ANFIS and (b) PSO-ANFIS.

Figure 1 outlines empirical data and the estimated yield values to represent predictive capability and the liability of the models. As can be observed, the concordance between the obtained and actual data regarding the efficiency of the models is exceptional.

**Figure 2:**
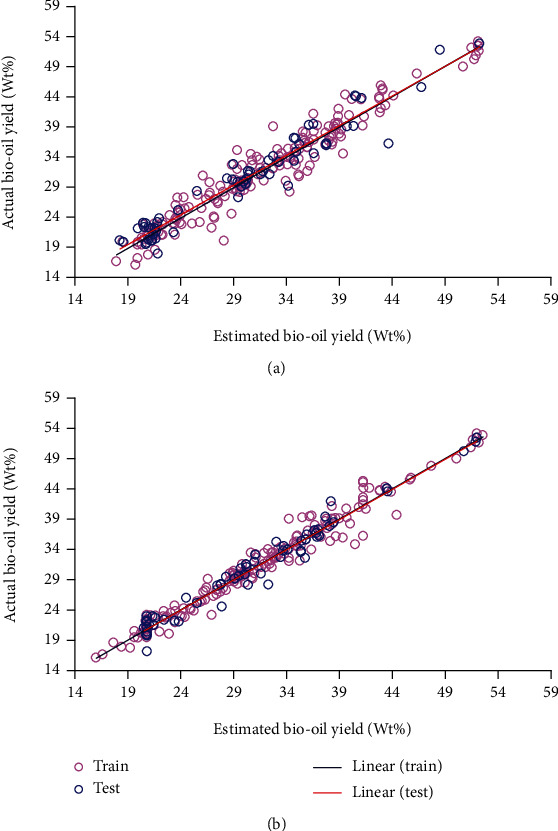
Cross-plot diagrams obtained using different models: (a) GA-ANFIS and (b) PSO-ANFIS.

Figure 2 displays the yield values obtained through models and experimental data. It shows a nearly straight line at the angle of 45° which confirms the ability of the model in producing accurate results. The displayed data indicates a higher level of *R*^2^ for the PSO-ANFIS model.

**Figure 3:**
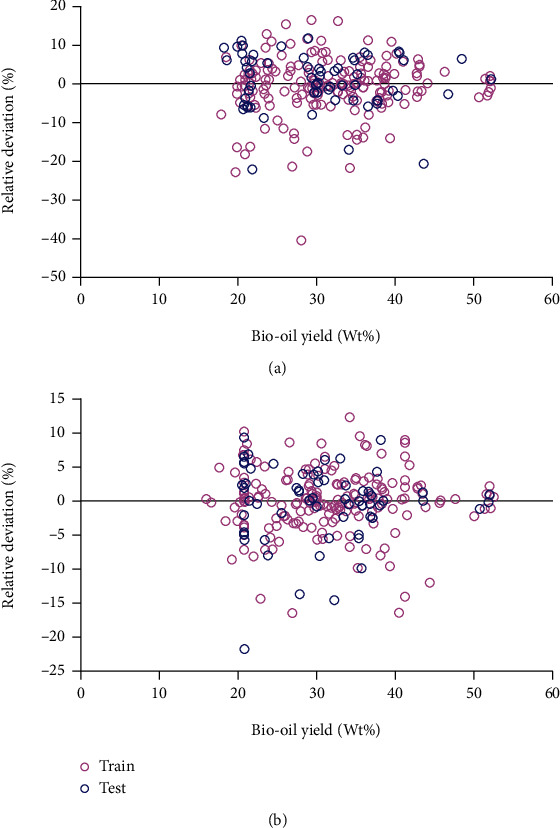
Relative derivation diagrams of (a) GA-ANFIS and (b) PSO-ANFIS models to evaluate their accuracy.

Figure 3 displays the relative derivations of both models. In estimating the yield values of diesel oils, the maximum absolute relative derivations of GA-ANFIS is 40% and of PSO-ANFIS is 23%. The corresponding values for biodiesel oils are 28 and 21, respectively. The statistical parameters indicate that the PSO-ANFIS model performs with the highest efficiency.

The present study, by comparing and assessing previous models developed by Tang et al. employing the same dataset on biodiesel oils [44], concluded that the PSO-ANFIS model performs more favorably in estimating the yield values. As seen in Table 2, this model boasts a more precise performance in estimation results than other models. The *R*^2^ and RMSE values for different models are as follows.

### 4.1. Sensitivity Analysis

In Equation (9), the relevancy factor examines the input parameters affecting yield values [50, 51].

(9) $$\begin{array}{c} r=\frac{\sum_{i=1}^{n}\left(X_{k,i}-{\overset{¯}{X}}_{k}\right)\left(Y_{i}-\overset{¯}{Y}\right)}{\sqrt{\sum_{i=1}^{n}{\left(x_{k,i}-{\overset{¯}{x}}_{k}\right)}^{2}\sum_{i=1}^{n}{\left(Y_{i}-\overset{¯}{Y}\right)}^{2}}}. \end{array}$$

**Figure 4:**
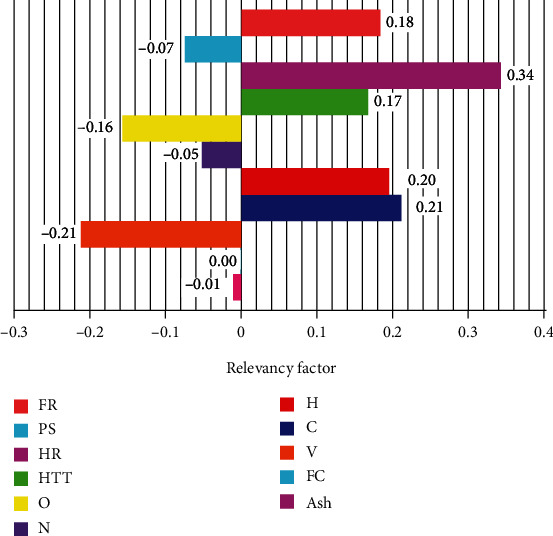
Sensitivity diagram on all input parameters affecting the output parameter.

The *i*th output is shown by *Y*_*i*_, the output average is shown by $\overset{¯}{Y}$, *X*_*k*,*i*_ denotes the *k*th input, and *x*_*k*_ denotes the input. The *r* value for each parameter is continually less than unity. The relevancy factor of biodiesel oil yield is shown in Figure 4. It can be observed that PS, O, N, and V have a negative effect on yield, and the effect of FR, HR, HTT, H, and C on the output is positive. This means that yield values of biodiesel oils are decreased by reducing the later parameters. In this figure, the relevancy factor of diesel oil yield is displayed. The largest impact on diesel oil yield is indicated for HR and the lowest for V.

## 5. Conclusion

The current paper is aimed at estimating the yield for diesel and biodiesel oils according to pyrolysis conditions and biomass compositions. For this purpose, the present study designed models using PSO-ANFIS and GA-ANFIS techniques and became the first to succeed in employing these techniques to estimate the output values. The PSO-ANFIS model boasts the most precise prognostication of target values. The raw data incorporated in this study was gathered from previous accredited researches, and statistical parameters (e.g., *R*^2^, %MRE, RMSE, MSE, and STD) in association with graphical valuations were employed in the testing and training stages of the model development. The findings attest to the high quality of performance and accuracy of the proposed PSO-ANFIS model. Therefore, it can be used to estimate output values with high accuracy in all related industries and processes.

## Figures and Tables

**Table 1:** The values of different statistical parameters obtained for the models.

Model	Phase	*R* ^2^	MRE (%)	MSE	RMSE	STD
GA-ANFIS	Train	0.937	5.077	4.244909156	2.0603	1.4186
	Test	0.937	5.693	4.432311766	2.1053	1.3085
	Total	0.937	5.231	4.291759808	2.1053	1.3910
PSO-ANFIS	Train	0.968	3.323	2.180267641	1.4766	1.0671
	Test	0.969	3.876	2.086124383	1.4443	0.9936
	Total	0.968	3.461	2.156731826	1.4443	1.0473

**Table 2:** Statistical comparison of the performance of different models in assessing the target values.

Model	*R* ^2^	RMSE
RF	0.87	3.05
MLR	0.284	7.96
PSO-ANFIS	0.968	1.4443

## Data Availability

The data used to support the findings of this study are provided within the article.
